# Supplementary material for: Glycometabolism regulates hepatitis C virus release
Source: PLoS Pathog. 2021 Jul 23;17(7):e1009746. doi: 10.1371/journal.ppat.1009746 (PMC8301660; doi:10.1371/journal.ppat.1009746)
Supplement: S1 Text — (DOC) [file ppat.1009746.s001.doc]

**Supplemental methods**

**Pseudotyped HCV infection**

HCV-pseudotyped particles (HCVpp) were generated as described previously [1]. Briefly, HEK293T cells were co-transfected with plasmids expressing HCV glycoproteins E1 and E2, retroviral core packaging component, and luciferase. The medium was refreshed at 6-hour post-transfection. Supernatants were collected and filtered at 72-hour post-transfection. For infection experiments, Huh7 cells were seeded in 96-well plates and pre-cultured with glucose or galactose medium for 12 hours, and then infected with HCVpp. The volume ratio of HCVpp inoclula to total culture medium was about 1:10 to minimize the potential influence of residual glucose in the HCVpp preparation. Three days after infection, the firefly luciferase activity was measured by using luciferase assay system according to the manufacturer’s instructions (Promega).

**Exosome isolation**

Exosomes were isolated from equivalent amounts of cell culture medium by differential ultracentrifugation as previously described [2]. For exosome isolation, the cell culture medium was changed to fresh glucose or galactose medium without fetal bovine serum 12 hours before sample collection. The culture medium was collected and subjected to sequential centrifugation as 10 min at 500 g to remove cells; 10 min at 2000 g to remove dead cells; 30 min at 10,000 g to remove cell debris and ultracentrifugation at 100,000 g for 70 min twice to wash and pellet exosomes. Exosome pellets were re-suspended in PBS for the following assays.

**Lactate, VLDL and total cholesterol detection**

Lactate assay kit (K627-100) was purchased from BioVision. VLDL ELISA kit (H249) and total cholesterol assay kit (A111-1) were purchased from Nanjing Jiancheng Bioengineering Institute. The lactate, VLDL and total cholesterol were measured according to the manufacturer’s instructions.

**shRNA knockdown**

The target sequences are as follows: PIKfyve, 5’-CCGGCCTTGGATTGTAACAATGGAA-3’; p38 sh1, 5’-CCTAGTAATCTAGCTGTGAAT-3’; p38 sh2, 5’-GCGGTTACTTAAACATATGAA-3’. The shRNA targeting irrelevant sequence was used as a nontarget control (shNC). For lentivirus production, HEK293T cells (5x10^5^ per well in 6-well plates) were co-transfected with 2 μg of pLKO.1-PIKfyve shRNA plasmid, together with 1.5 μg of packaging plasmid pVSVg (AddGene) and psPAX2 (AddGene). The culture supernatants were harvested at 48-hour post-transfection and filtered through 0.45 μm-pore-size filters. For PIKfyve knockdown experiment, Huh7 cells were first infected with HCV at MOI of 2 for one day, and then transduced with shRNA-lentiviruses for 2 days. For p38 knockdown experiment, Huh7 cells were transduced with shRNA-lentiviruses for 2 days, then seeded onto a 12-well plate for infection of HCV at MOI of 2 for 3 days.

**MKK6EE ectopic expression**

The open reading frame of MKK6 was amplified from cDNA of Huh7 cells and cloned into plvx-puro plasmid (AddGene). FLAG tag was linked to N-terminal sequence of MKK6, the double mutations of S207E/T211E were introduced by ClonExpress MultiS One Step Cloning Kit (Vazyme). For lentivirus production, HEK293T cells (5x10^5^ cells per well in 6-well plates) were co-transfected with 2 μg of empty plvx-puro or MKK6EE-overexpressing plasmid, together with 1.5 μg of packaging plasmid pVSVg (AddGene) and psPAX2 (AddGene). The culture supernatants were harvested at 48-hour post-transfection and filtered through 0.45 μm-pore-size filters. Huh7 cells cultured in galactose medium were infected with HCV at MOI of 2 for 24 hours, then transduced with lentiviruses expressing MKK6EE for 72 hours.

**Quantitative RT-PCR**

For RT-PCR assay, the total RNA isolation from cells or cell culture medium was performed by using TRNzol (TIANGEN Biotech). For the RNA isolation from the supernatants, the same amount of naïve Huh7 total cellular RNAs were added to culture supernatants as an internal control to normalize the variation of RNA extraction. The cDNA synthesis was using ReverTra Ace qPCR RT Kit (Toyobo), and the real-time PCR was performed by using SYBR Green Realtime PCR Master Mix (Toyobo) according to the manufacturer’s instructions. The primer sequences are as follows: HCV forward: 5’-TCTGCGGAACCGGTGAGTA-3’, HCV reverse: 5’-TCAGGCAGTACCACAAGGC-3’; SREBP1 forward: 5’-ACAGTGACTTCCCTGGCCTAT-3’, SREBP1 reverse: 5’-GCATGGACGGGTACATCTTCAA-3’; SEBEP2 forward: 5’-AACGGTCATTCACCCAGGTC-3’, SEBEP2 reverse: 5’-GGCTGAAGAATAGGAGTTGCC-3’; SCD1 forward: 5’-GCCCCTCTACTTGGAAGACGA-3’, SCD1 reverse: 5’-AAGTGATCCCATACAGGGCTC-3’; ELOVL6 forward: 5’-AACGAGCAAAGTTTGAACTGAGG-3’, ELOVL6 reverse: 5’-TCGAAGAGCACCGAATATACTGA-3’; ACACA forward: 5’-CATGCGGTCTATCCGTAGGTG-3’, ACACA reverse: 5’-GTGTGACCATGACAACGAATCT-3’. PIKfyve forward: 5’-GAACAGCAGCCTTTGAGTGGA-3’, PIKfyve reverse: 5’- GGTGTGGGTGACCTAACAGAC-3’; β-Actin forward: 5’-AGTGTGACGTGGACATCCGCAAAG-3’, β-Actin reverse: 5’-ATCCACATCTGCTGGAAGGTGGAC-3’. The expression of target genes was normalized to the expression of β-Actin.

**Western blot and immunofluorescence**

An equal amount of protein (20~40 μg) from each sample in loading buffer were subjected to electrophoresis in 10%~12.5% SDS-polyacrylamide gel and transferred onto PVDF membranes (Millipore) for Western blot assay.

Immunofluorescence microscopy was used to identify the expression and subcellular localization of CD63 in Huh7 cells. Nuclei were stained with Hoechst 33342 (Invitrogen).

The primary antibodies for Western blot and immunofluorescence assay include CD63 (Santa Cruz), LAMP1 (Abcam), SREBP2 (Abcam), ApoE (Millipore), β-actin (Abgent), p-p38 (CST), p38 (CST) and DENV (Abcam). The anti-ZIKV NS3 protein antibody is customized. The mouse anti-HCV Core, NS3 and NS5A antibodies were generated by Abmart (Shanghai). The second antibodies include goat anti-rabbit IgG-HRP (Santa Cruz), HRP goat anti-mouse (Jackson), Alexa Fluor 555 donkey anti-mouse IgG (H+L) (Invitrogen) and Alexa Fluor 488 donkey anti-rabbit IgG (H+L) (Invitrogen).

**Flow cytometry analysis**

Huh7 cells were cultured in glucose and galactose medium for 48 hours respectively. Then cells were fixed and permeabilized with permeabilization buffer (eBioScience) and stained with control mouse IgG antibody (Santa Cruz) or CD63 antibody (Santa Cruz). Incubation for 30 min at room temperature. Data was acquired by Calibur (BD Biosciences) and analyzed using FlowJo software.

**References**

1. Xiang Y, Tang JJ, Tao W, Cao X, Song BL, Zhong J. Identification of Cholesterol 25-Hydroxylase as a Novel Host Restriction Factor and a Part of the Primary Innate Immune Responses against Hepatitis C Virus Infection. J Virol. 2015;89(13):6805-16. Epub 2015/04/24. doi: 10.1128/JVI.00587-15. PubMed PMID: 25903345; PubMed Central PMCID: PMCPMC4468479.

2. Li P, Kaslan M, Lee SH, Yao J, Gao Z. Progress in Exosome Isolation Techniques. Theranostics. 2017;7(3):789-804. Epub 2017/03/04. doi: 10.7150/thno.18133. PubMed PMID: 28255367; PubMed Central PMCID: PMCPMC5327650.
