# Supplementary figures and images for: Glycometabolism regulates hepatitis C virus release
Source: PLoS Pathog. 2021 Jul 23;17(7):e1009746. doi: 10.1371/journal.ppat.1009746 (PMC8301660; doi:10.1371/journal.ppat.1009746)

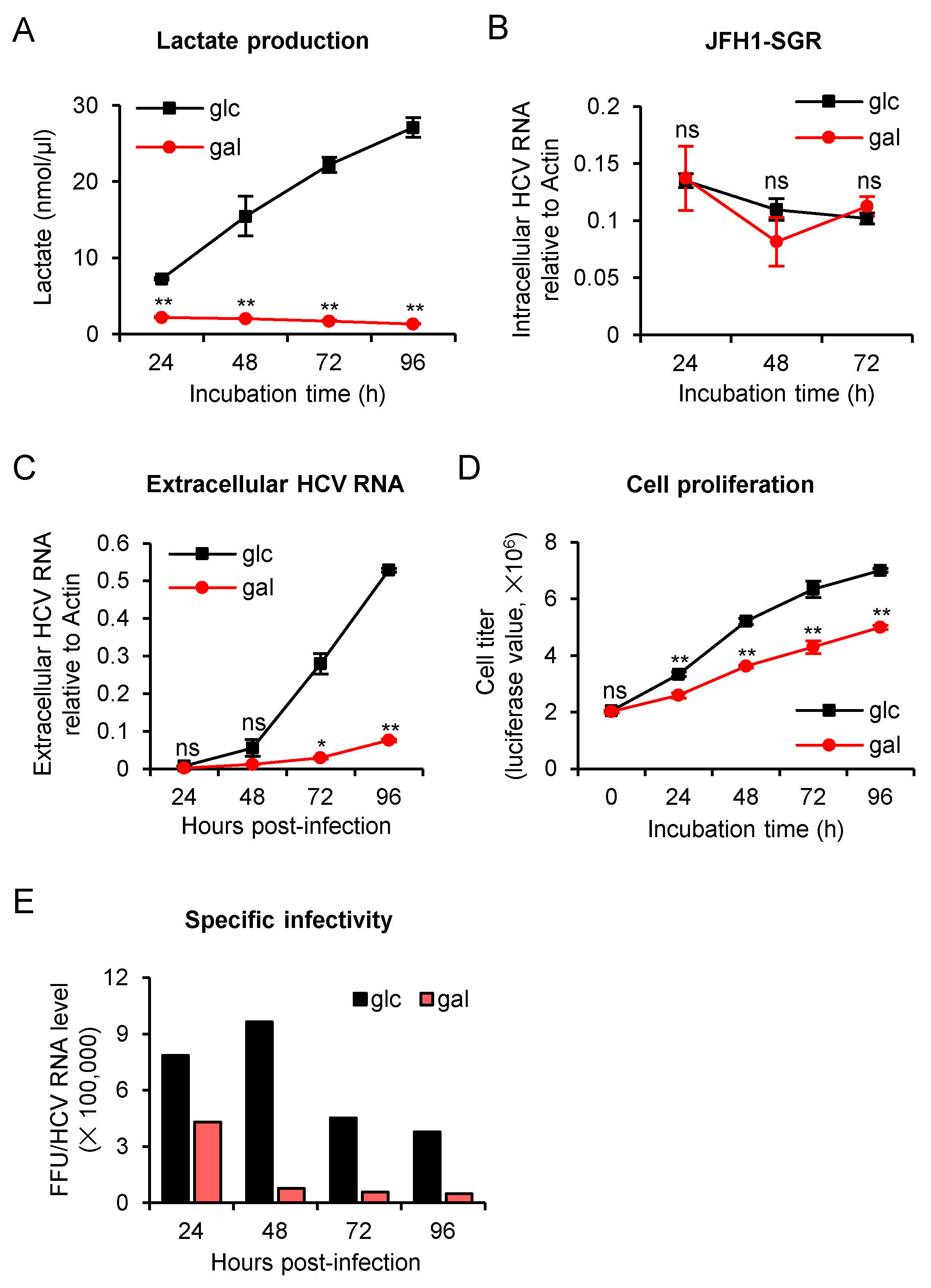

Supplement: S1 Fig — (A) Lactate production assay. Huh7 cells were cultured in glucose or galactose medium, and quantified at 24-, 48-, 72- and 96-hour after cell seeding by using the lactate colorimetric assay kit. (B) RT-qPCR analysis of HCV RNA levels in Huh7 cells harboring JFH1 subgenomic replicon (SGR) that had been cultured in glucose or galactose medium for 24, 48 and 72 hours. (C) Huh7 cells that had been cultured in glucose or galactose medium for 12 hours were infected with HCV at MOI of 2. The extracellular HCV RNA levels in the culture supernatants were examined at 24-, 48-, 72- and 96-hour post-infection by RT-qPCR. (D) Specific infectivity was calculated from Figs 1F and S1C. (E) Cell titer assay. Huh7 cells were cultured in glucose or galactose medium, and quantified at 0-, 24-, 48-, 72- and 96-hour after cell seeding by using the CellTiter-Glo cell viability assay kit. Data were presented as the mean ± standard deviation (error bars) of triplicates (A, B, C, E). glc: glucose medium; gal: galactose medium. (TIFF) [file ppat.1009746.s002.tiff]

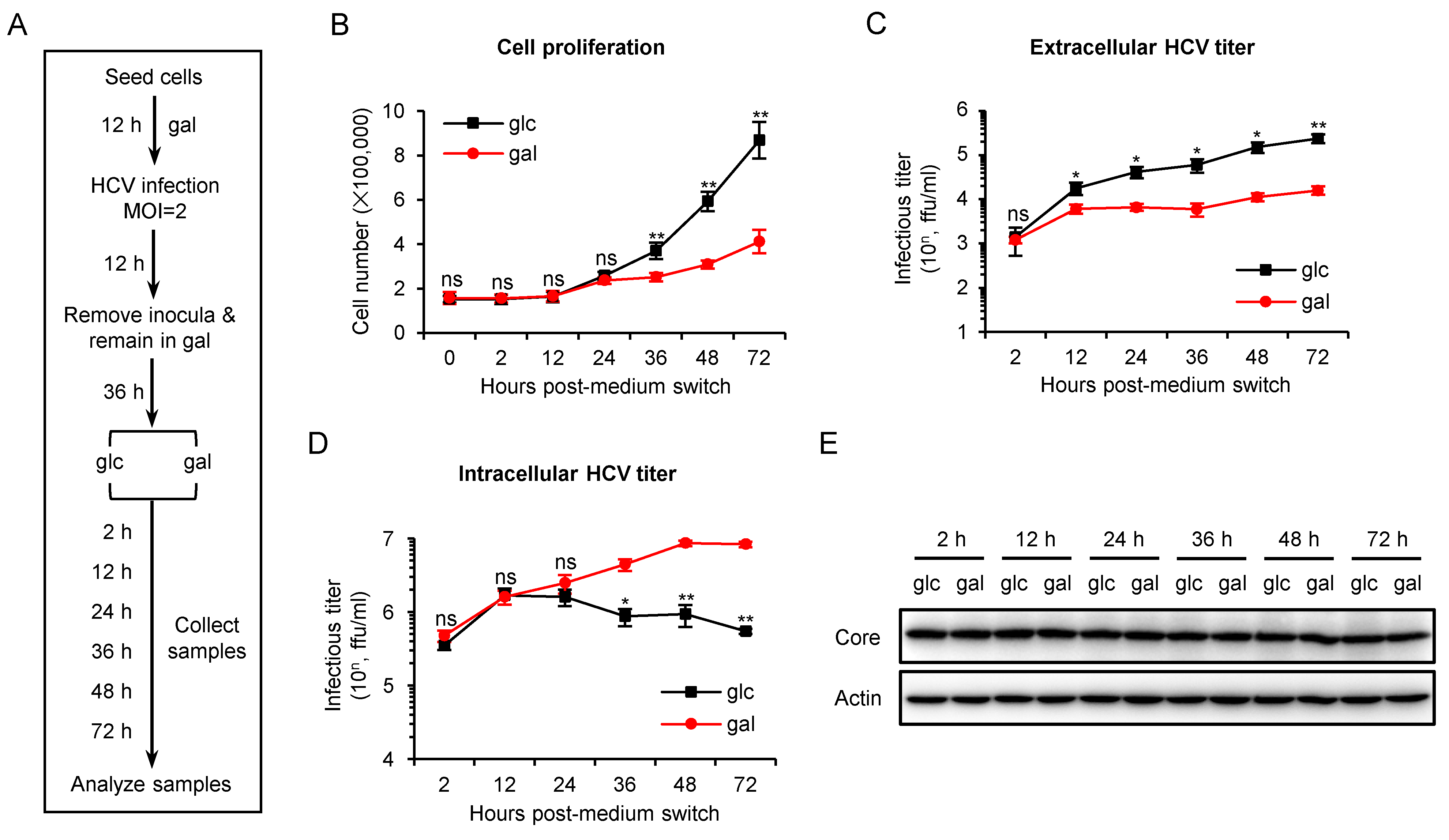

Supplement: S2 Fig — (A) Schematic representation of the medium switch experiment. (B-E) Huh7 cell samples were collected at 0-, 2-, 12-, 24-, 36-, 48- and 72-hour post-medium switch. The cell number (B), extracellular titer (C), intracellular titer (D) and viral Core protein (E) were determined. Data were presented as the mean ± standard deviation (error bars) of triplicates. glc: glucose medium; gal: galactose medium. (TIFF) [file ppat.1009746.s003.tiff]

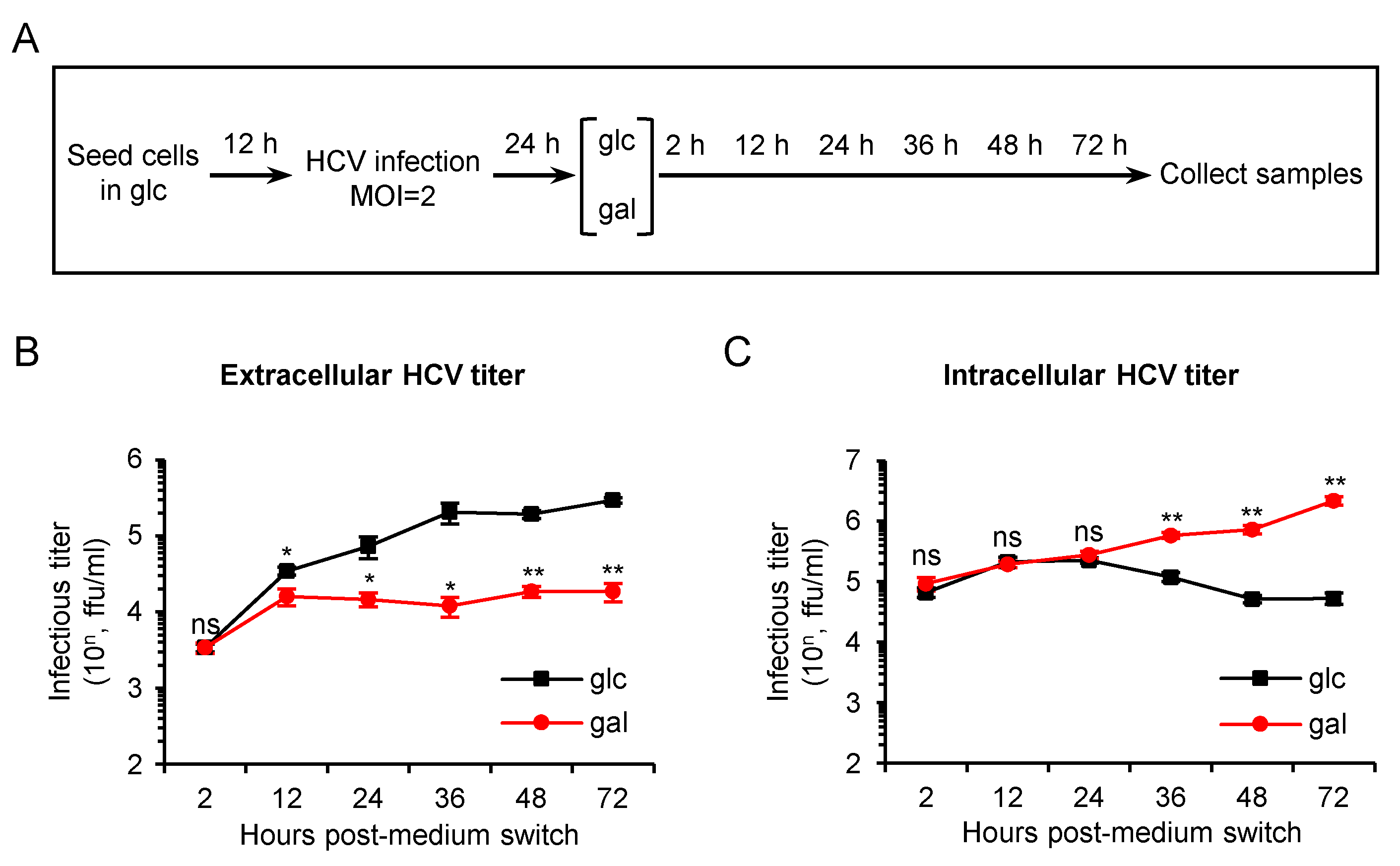

Supplement: S3 Fig — (A) Schematic of the medium switch experiment. (B-C) Kinetics of extracellular and intracellular HCV titers following medium switch. Huh7 cells were infected with HCV at MOI of 2 in glucose medium. The extracellular and intracellular HCV titers were measured at 2-, 12-, 24-, 36-, 48- and 72-hour after the medium switch. Data were presented as the mean ± standard deviation (error bars) of triplicates. glc: glucose medium; gal: galactose medium. (TIFF) [file ppat.1009746.s004.tiff]

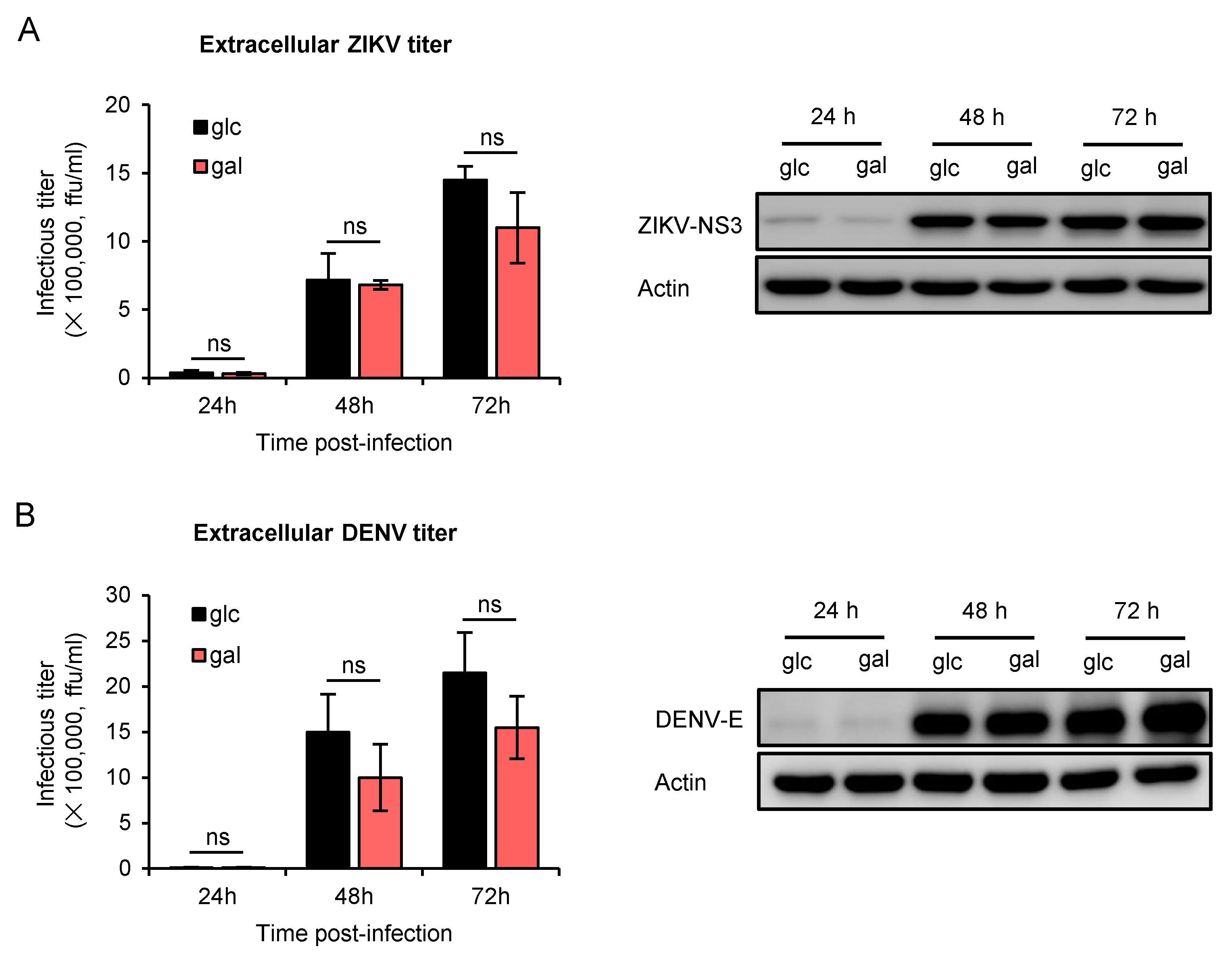

Supplement: S4 Fig — Huh7 cells cultured in glucose and galactose medium were infected by ZIKV at moi of 0.05 (A) or DENV at moi of 0.01 (B). Extracellular virus titer and intracellular ZIKV-NS3 or DENV-E proteins at 24-, 48- and 72-hour post-infection were examined respectively. Data were presented as the mean ± standard deviation (error bars) of triplicates. glc: glucose medium; gal: galactose medium. (TIFF) [file ppat.1009746.s005.tiff]

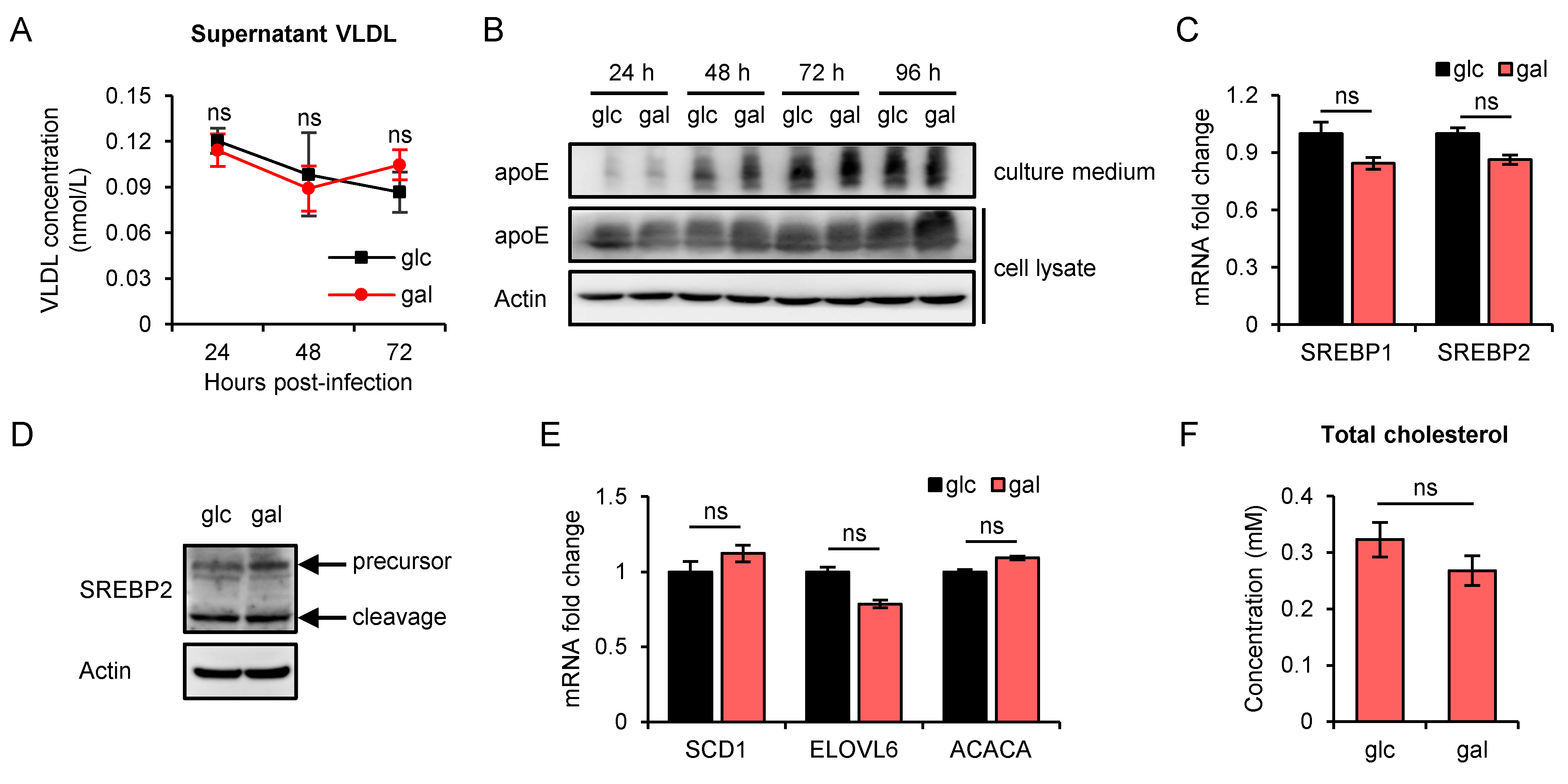

Supplement: S5 Fig — (A) Huh7 cells that had been cultured in glucose or galactose medium for 12 hours were infected with HCV at MOI of 2. The VLDL levels in the culture supernatants were measured at 24-, 48- and 72-hour post-infection. (B) Huh7 cells were cultured in glucose or galactose medium for 24, 48, 72 and 96 hours. Intracellular and extracellular ApoE levels were examined by Western blot. (C-E) The mRNA (C) and protein (D) levels of SREBP1 or SREBP2, as well as their target gene SCD1, ELOVL6 and ACACA mRNA levels (E) in Huh7 cells cultured in glucose or galactose medium for 72 hours. (F) Total cholesterol levels were measured in Huh7 cells cultured in glucose or galactose medium for 72 hours. Data were presented as the mean ± standard deviation (error bars) of triplicates. glc: glucose medium; gal: galactose medium. (TIFF) [file ppat.1009746.s006.tiff]

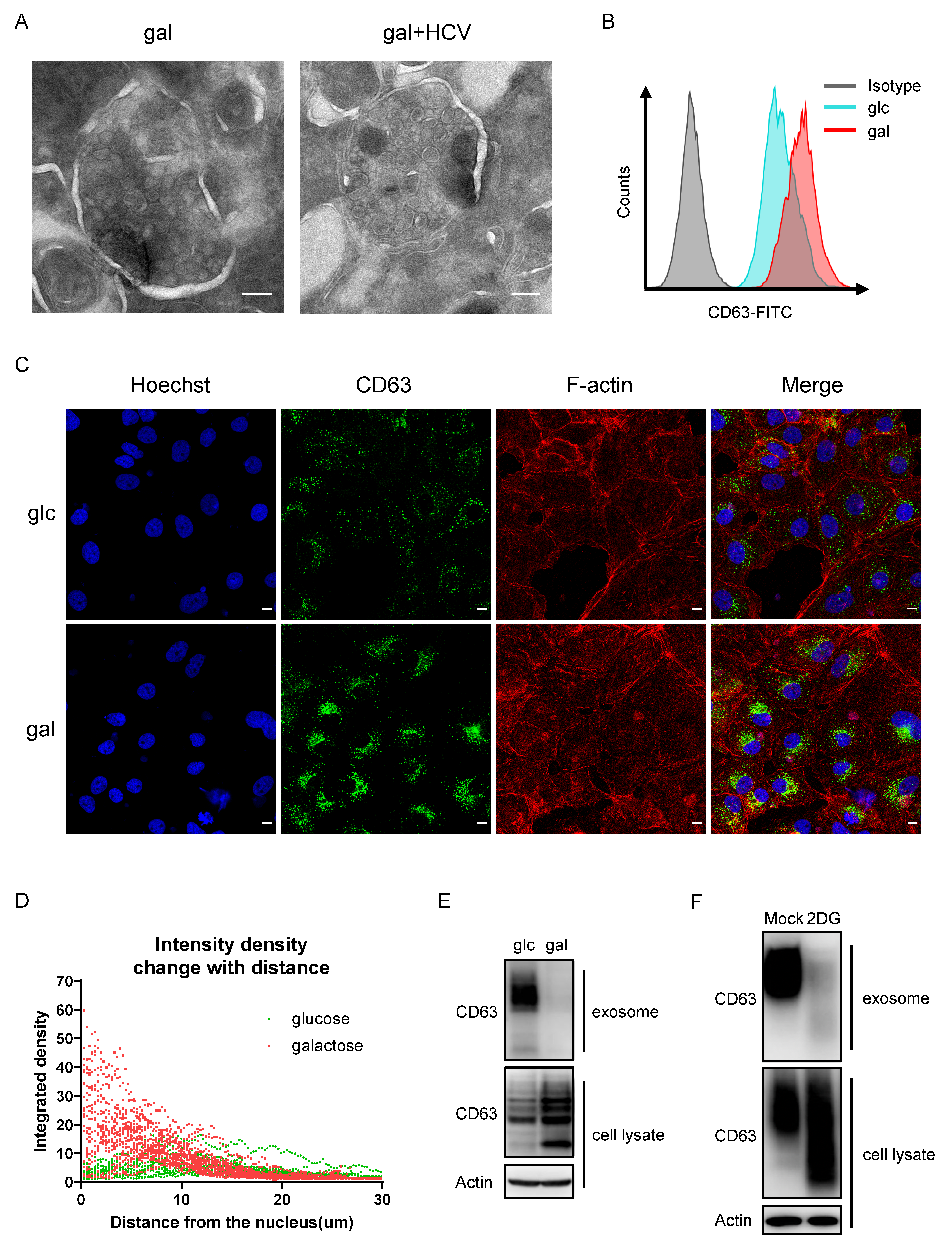

Supplement: S6 Fig — (A) Representative transmission electron microscopic images of MVB and intraluminal vesicles (ILVs) in the galactose medium-cultured Huh7 cells with or without HCV infection (bar 100 nm). (B) Flow cytometry analysis of CD63 expression in the Huh7 cells cultured in glucose or galactose medium. (C) Confocal immunofluorescent images of CD63 (green) and F-actin (red) in Huh7 cells that had been cultured in glucose or galactose medium for 48 hours. Nuclei were stained with Hoechst (blue). (D) Quantification of CD63 intensity and distance from the nucleus, n (cell number) > = 10. (E) Western blot of the exosome-associated or intracellular CD63 in Huh7 cells cultured in glucose or galactose medium for 48 hours. (F) Western blot of the exosome-associated or intracellular CD63 in Huh7 cells cultured in glucose medium supplemented with 4 mM 2DG for 72 hours. glc: glucose medium; gal: galactose medium. (TIFF) [file ppat.1009746.s007.tiff]

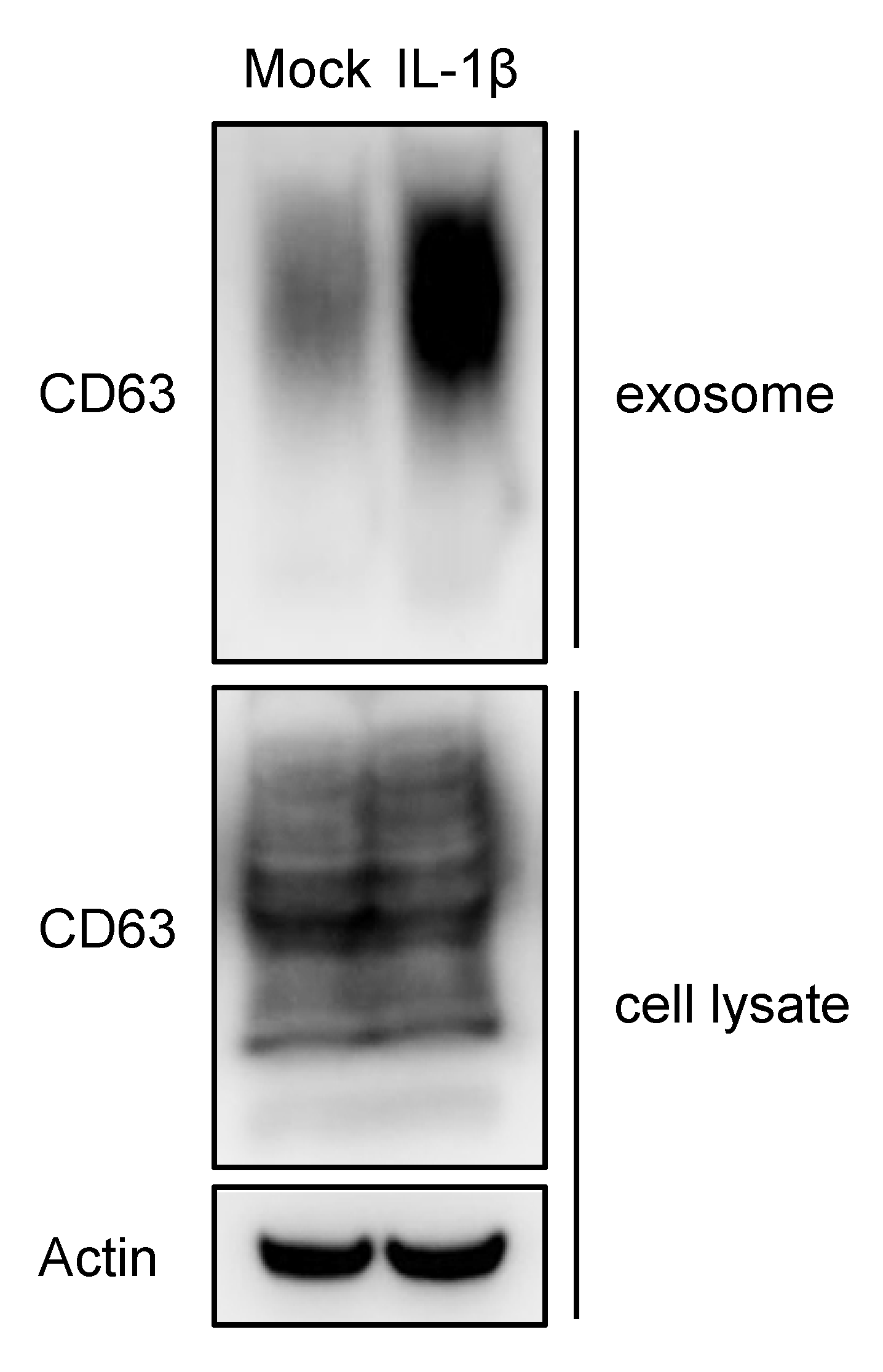

Supplement: S7 Fig — Western blot of the exosome-associated or intracellular CD63 in Huh7 cells cultured in galactose medium treated with or without IL-1β (10 ng/ml) for 48 hours. (TIFF) [file ppat.1009746.s008.tiff]
