# Supplementary material for: Glycometabolism regulates hepatitis C virus release
Source: PLoS Pathog. 2021 Jul 23;17(7):e1009746. doi: 10.1371/journal.ppat.1009746 (PMC8301660; doi:10.1371/journal.ppat.1009746)
Supplement: Attachment — Submitted filename: Comments_MS.docx [file ppat.1009746.s009.docx]

This study by Yu *et al.* explores the effect of glycolysis versus oxidative phosphorylation on HCV life cycle. The authors culture human hepatoma HuH-7 cells in galactose-containing medium to switch energy production from glycolysis to oxidative phosphorylation and show that this inhibits the release of mature virus particles into culture medium. Electron microscopy of the infected cells revealed virus particles being trapped in multivesicular bodies (MVBs), which accumulated to higher numbers in galactose-treated cells as compared to those cultured in glucose, suggesting metabolism-dependent release of MVBs (and therefore of HCV) from infected cells. Chemical or genetic manipulation of cells aimed at forcing the release of MVBs restored HCV exodus from the cells. Furthermore, cell-to-cell transmission of HCV does not seem to be affected by the metabolic state of cells. Because oxidative phosphorylation is the predominant glycometabolic pathway in hepatocytes *in vivo*, the authors propose an attractive hypothesis that HCV exploits this metabolic state of cells to mainly spread through cell-to-cell transmission and thereby evade host immune surveillance.

It is an elegant piece of work, clearly presented, and strongly supported by the data. The experiments are well controlled and precisely described. This reviewer has no major concerns, but do have problems with how the authors interpreted Fig. 3. Below I highlight my concerns and provide some suggestions to improve the quality of the manuscript.

1. There is inconsistency in the y-axes of graphs across figures and figure panels. For example, the y-axis in Fig. 1A is formatted in scientific, but it is formatted as a multiple of 100,000 in Fig. 1H. This pattern applies to all figures. Please choose one format and stick with it to facilitate the interpretation of results.
2. In Fig. 1B, intracellular HCV RNA is presented as relative to actin. The numbers on the y-axis look strange and are hard to believe. It appears as if the amount of HCV RNA in cells is only 6-fold higher than that of actin, which is probably not the case. Also, the graph gives an impression that there was hardly any viral RNA at 4 days post-infection when there should in fact be tens of thousands of RNA copies inside a cell. It would be better to present your data as RNA copies per microgram of total RNA.
3. Fig. S1C shows extracellular HCV RNA relative to actin? What does this mean? Probably a mistake?
4. The authors didn’t see any effect of galactose on the entry of HCV pseudoparticles (Fig. 1C). Were the pseudoparticles generated in glucose-containing medium or did the authors use purified particles. Please explain in detail how the experiment was being performed and also describe the possible effect of glucose in the HCVpp prep on virus entry.
5. The titer of intracellular infectious HCV particles is usually (almost always) significantly lower than that of extracellular infectious virions. In Fig. 1F and 1G, intracellular titers in glucose-treated cells (~400,000 per ml at 24 hpi) appear to be much higher than extracellular titer (~ 5,000 per ml at 24 hpi). How to explain this? Also, what is the authors’ explanation for intracellular virus titers remaining unchanged from 24h to 96h in glucose-treated cells (Fig. 1G)?
6. This reviewer doesn’t agree with the conclusions the authors draw from Fig. 3B and 3C. I don’t see more CD63 in fraction 4 compared to fraction 8 in glucose-treated cells. Similarly, the distribution of E2, core, and infectious particles across various fractions look similar for both media. The authors should repeat the experiment and provide quantitative data to prove their point.
7. In Fig. 4C, the authors show that knocking down PIKFYVE restored HCV release from galactose-treated cells. What was the effect of this knockdown in glucose-treated cells? This is an important experiment given that the chemical inhibition of PIKFYVE seems to cause equal inhibition of HCV replication in both media.
8. The authors should test how the treatment of galactose-treated cells with pro-inflammatory cytokines affected the abundance of MVBs in the cells.

**Minor comments:**

1. Abstract: line 9. Please add “treatment with” before pro-inflammatory cytokines.
2. Author summary: line 1. I don’t think it is fair to call HCV a noncytopathic virus. It does inflict damage to the cells, although the damage might be less obvious compared to some lytic viruses.
3. Author summary: line 7. Please add “because” before hepatoma-derived cell lines
4. Author summary: lines 11-14. These lines describe results that are not directly relevant to glucose vs. galactose. Please describe what you observed and learnt about the effect of glycometabolism on HCV release and spread. To further clarify this point, this study is not the first to provide an evidence that MVB are intracellular reservoir of nascent HCV virions, nor is this the first description of distinct mechanisms behind cell-to-supernatant vs. cell-to-cell spread. In contrast, this story showed that intracellular accumulation of MVB is increased in galactose medium, and that the virus mode of spread is affected by the glycometabolic state of cells.
5. Introduction: Page 1, line 16: Please replace “viral Core proteins” with “the viral core protein”.
6. Introduction: Page 2, lines 9-11. This sentence is redundant with the statement made on lines 3-4 of this page.
7. Results: Page 2, line 24. Please replace “remained” with “left”.
8. Please add page numbers to your manuscript so it is easy to point out errors/edits.
