# Supplementary material for: Glycometabolism regulates hepatitis C virus release
Source: PLoS Pathog. 2021 Jul 23;17(7):e1009746. doi: 10.1371/journal.ppat.1009746 (PMC8301660; doi:10.1371/journal.ppat.1009746)
Supplement: Attachment — Submitted filename: Editor and Reviewer comments_response.doc [file ppat.1009746.s010.doc]

Dear editors and reviewers,

We are very grateful for the constructive questions and suggestions from the editors and reviewers about our manuscript submitted to PLOS Pathogens (Manuscript Number: PPATHOGENS-D-21-00597). As requested, we have addressed all the comments as below. All changes can be tracked in the revised manuscript.

**Reviewer #1:**

**Major issues:**

Q1. Given the primary hepatocytes likely use oxidative phosphorylation for energy metabolism, is it possible to manipulate that to perhaps increase infection/release efficiency of HCV in these cells? Are there ways to shift the balance more towards aerobic glycolysis? A way could be to manipulate PKM2 etc?

A. Thanks so much for your insightful question. Primary hepatocytes are not readily infected by HCV, and the efficiency of infection varies among different labs depending on the source of the primary cells. We previously tried commercially available primary hepatocytes for HCV infection. The infection was not robust, and no progeny virions can be detected. Therefore, we are unable to determine the impact of glycometabolism on HCV release in primary hepatocytes. There are several ways to shift the metabolism toward aerobic glycolysis in the primary cells, for example, treatment with EGF or TNFα, manipulating PKM2 dimeric formation with pTyr, and inhibiting oxidative phosphorylation to boost glycolysis. We have added some discussion on this point in the revised manuscript (tracked version page 16 line 349 to 354).

Q2. The precise mechanism by which the galactose switch traps particles in the MVB is not understood. The authors speculate that a general shift of outbound versus inbound traffic is responsible, maybe they should examine this shift and demonstrate that it indeed occurs with experiment in this model system.

A. We agree with the reviewer. To address this question, we performed quantitative confocal immunofluorescence of CD63 by measuring its distance from the nuclei. As presented in new Fig S6C-D, these results showed that CD63 was dispersed in the cytoplasm in glucose-cultured cells, but mainly concentrated near the nuclei in the galactose-cultured cells. These results suggested that the MVBs are more likely to move inward in galactose medium-cultured cells and to move outward in glucose medium-cultured cells. In addition, inhibition of the MVB-lysosome fusion in the galactose-cultured cells could restore the release of trapped HCV (Fig 4), suggesting that HCV are likely to be trapped within MVBs of the galactose medium-cultured cells and can be reversed by blocking inbound traffic of MVBs.

**Minor issues:**

The p38 part appears to be isolated and a bit underdeveloped. How does that connect with the traffic shifting hypothesis? Is the phosphorylation change a cause or result of the shift? This point should be at least addressed in discussion if can’t be easily addressed with experiments.

A. Our results showed that p38 phosphorylation and HCV release were reduced in Huh7 cells cultured in galactose medium or treated with glycolysis blocker 2DG, but enhanced by IL-1β treatment (Fig 1, 5 and 6), hinting a correlation between p38 phosphorylation and HCV release. Furthermore, we showed that HCV release can be reduced by a chemical inhibitor or shRNA targeting p38 (Fig 6D-I), suggesting that p38 plays a role in regulating HCV release and endosome tracking. We tend to think that the p38 phosphorylation is the cause rather than result because a constitutively active form of MKK6 known to catalyze p38 phosphorylation can promote HCV release in the galactose-cultured cells (Fig 6J-L). It has also been reported that p38 inhibition can downregulate expression levels of several genes related to exosome biogenesis and secretion, such as RAB27A, RAB27B, RAB11A andSMPD3, suggesting the p38 signaling pathway might be involved in regulating endosome tracking and exosome secretion. We have added a few lines of discussion on this point in the revised manuscript (tracked version page 18 line 404 to 413).

**Reviewer #2:** This reviewer has no major concerns, but do have problems with how the authors interpreted Fig. 3. Below I highlight my concerns and provide some suggestions to improve the quality of the manuscript.

Q1. There is inconsistency in the y-axes of graphs across figures and figure panels. For example, the y-axis in Fig. 1A is formatted in scientific, but it is formatted as a multiple of 100,000 in Fig. 1H. This pattern applies to all figures. Please choose one format and stick with it to facilitate the interpretation of results.

A. Thanks a lot for pointing this out. We have reformatted some figures to have the same format for the y-axes of all figures (please see Figure 1A, 1B, 1F, 5E, 5F, 7A, S2C, S2D, S3B and S3C in the revised manuscript).

Q2. In Fig. 1B, intracellular HCV RNA is presented as relative to actin. The numbers on the y-axis look strange and are hard to believe. It appears as if the amount of HCV RNA in cells is only 6-fold higher than that of actin, which is probably not the case. Also, the graph gives an impression that there was hardly any viral RNA at 4 days post-infection when there should in fact be tens of thousands of RNA copies inside a cell. It would be better to present your data as RNA copies per microgram of total RNA.

A. Thanks a lot for pointing this out. We performed this experiment by using a low moi HCV infection (moi=0.01). That’s why HCV RNA levels remained low until day 4 post-infection. We have changed the y-axis to a logarithmic scale which illustrates better HCV RNA at low levels.

Q3. Fig. S1C shows extracellular HCV RNA relative to actin? What does this mean? Probably a mistake?

A. Thanks so much for the question. To normalize the variations in RNA extraction from culture supernatants, we added to the supernatants with the same amount of total cellular RNA extracted from naïve Huh7 cells. This would allow us to use cellular actin mRNA as an internal control in qPCR. We have added this information in the supplemental methods (page 5 line 84 to 87).

Q4. The authors didn’t see any effect of galactose on the entry of HCV pseudoparticles (Fig. 1C). Were the pseudoparticles generated in glucose-containing medium or did the authors use purified particles. Please explain in detail how the experiment was being performed and also describe the possible effect of glucose in the HCVpp prep on virus entry.

A. This is a good point. We did not purify the HCV pseudoparticles (HCVpp). However, we don’t think the residual glucose in the HCVpp viral supernatants would affect the result for the following reasons. First, HCVpp was collected 72 hours after the transfection, and glucose has been mostly consumed by the cells at this time point. Second, only small amount of HCVpp was inoculated to Huh7 cells that are cultured in a large volume of either glucose or galactose medium (about 1:10 ratio in volume). Therefore, the residual glucose in the viral inocula should not change the actual final concentration of glucose in the medium. Third, HCVcc virus stock was also prepared in the glucose-containing medium, and HCVcc infection experiments were performed in a similar fashion as the HCVpp infection. We have added this information in the supplemental methods of the revised manuscript (page 3 line 38 to 40).

Q5. The titer of intracellular infectious HCV particles is usually (almost always) significantly lower than that of extracellular infectious virions. In Fig. 1F and 1G, intracellular titers in glucose-treated cells (~400,000 per ml at 24 hpi) appear to be much higher than extracellular titer (~ 5,000 per ml at 24 hpi). How to explain this? Also, what is the authors’ explanation for intracellular virus titers remaining unchanged from 24h to 96h in glucose-treated cells (Fig. 1G)?

A. Thanks so much for the questions. To determine the intracellular HCV titers, approximately 1x10^6^ cells were lysed in a small volume (50 μl) of complete medium by freezing-thaw cycles, and the titers were measured and expressed as foci formation units per milliliter (ffu/ml). Therefore, the high intracellular titer was due to the small volume of medium used to lyse the cells. We have described the method in detail in the revised manuscript (tracked version page 20 line 445).

For the second question, we think that the intracellular virus titers remaining unchanged from 24 to 96h in the glucose-treated cells is likely because the level of intracellular virions quickly reaches a plateau after a high MOI infection and then maintains at a relatively stable level.

Q6. This reviewer doesn’t agree with the conclusions the authors draw from Fig. 3B and 3C. I don’t see more CD63 in fraction 4 compared to fraction 8 in glucose-treated cells. Similarly, the distribution of E2, core, and infectious particles across various fractions look similar for both media. The authors should repeat the experiment and provide quantitative data to prove their point.

A. We have followed the reviewer’s suggestion to repeat this experiment and then to quantify the CD63 levels in fractions 4 and 8. As presented in new Fig 3D of the revised manuscript, the results demonstrated a shift of the CD63 distribution toward fraction 8 in the galactose-cultured cells. We agree with the reviewer that there was no apparent difference in the distribution of E2, core and infectious particles between the two treatments. As most intracellular viral proteins are not assembled into viral particles, their distributions should reflect the subcellular localization of these proteins rather than virions. As HCV replication is not affected by glycometabolism, we do not expect a change in their distribution.

Q7. In Fig. 4C, the authors show that knocking down PIKFYVE restored HCV release from galactose-treated cells. What was the effect of this knockdown in glucose-treated cells? This is an important experiment given that the chemical inhibition of PIKFYVE seems to cause equal inhibition of HCV replication in both media.

A. We agree with the reviewer. We performed this PIKfyve knocking-down experiment in glucose medium-cultured cells. The results have been presented as new Fig 4C of the revised manuscript.

Q8. The authors should test how the treatment of galactose-treated cells with pro-inflammatory cytokines affected the abundance of MVBs in the cells.

A. We followed the reviewer’s suggestion to determine the impact of IL-1β on the extracellular and intracellular CD63 levels in the galactose-cultured cells. As showed in new Fig S7, IL-1β significantly promotes the exosomal CD63 release, while has no significant effect on the abundance of intracellular CD63. We have added this new result in the revised manuscript (Fig S7).

Minor comments:

1. Abstract: line 9. Please add “treatment with” before pro-inflammatory cytokines.

A. We have corrected this error in the revised manuscript.

2. Author summary: line 1. I don’t think it is fair to call HCV a noncytopathic virus. It does inflict damage to the cells, although the damage might be less obvious compared to some lytic viruses.

A. We have deleted the expression of “noncytopathic”.

3. Author summary: line 7. Please add “because” before hepatoma-derived cell lines.

A. We have added “because” before “hepatoma-derived cell lines”.

4. Author summary: lines 11-14. These lines describe results that are not directly relevant to glucose vs. galactose. Please describe what you observed and learnt about the effect of glycometabolism on HCV release and spread. To further clarify this point, this study is not the first to provide an evidence that MVB are intracellular reservoir of nascent HCV virions, nor is this the first description of distinct mechanisms behind cell-to-supernatant vs. cell-to-cell spread. In contrast, this story showed that intracellular accumulation of MVB is increased in galactose medium, and that the virus mode of spread is affected by the glycometabolic state of cells.

A. We have rephrased the statement.

5. Introduction: Page 1, line 16: Please replace “viral Core proteins” with “the viral core protein”.

A. We have made the correction.

6. Introduction: Page 2, lines 9-11. This sentence is redundant with the statement made on lines 3-4 of this page.

A. We have deleted the redundant sentence mentioned above.

7. Results: Page 2, line 24. Please replace “remained” with “left”.

A. We have replaced “remained’ with “left”.

8. Please add page numbers to your manuscript so it is easy to point out errors/edits.

A. The page numbers have been added in the revised manuscript.

Reviewer #3: This is a well-planned and carried out study, and the manuscript is very well written. The topic is important, and the finding are interesting and justified. This reviewer has the following questions/concerns.

Q1. In Suppl Fig S1, can a comparison of glycometabolic pathways between Huh7 cells and primary hepatocytes by included? One could also imagine comparing the impacts of galactose and glucose on infectious virus release from such primary cells, although replication efficiently will limit the utility of this system.

A. Thanks so much for the insightful suggestion. It has been reported that compared to primary human hepatocytes, Huh7 cells takes up more glucose and secret much more lactate, suggesting that glycolysis is much stronger in Huh7 cells (doi: 10.1002/elps.201300228). As the reviewer rightfully pointed out, primary hepatocytes are not readily infected by HCV. We previously tried commercially available primary hepatocytes for HCV infection. The infection was not robust, and no progeny virions can be detected. Therefore, we are unable to determine the impact of glycometabolism on HCV release in primary hepatocytes. Nevertheless, we have added some discussion on this point in the revised manuscript (tracked version page 16 line 349 to 354).

Q2. While the impact of galactose on infectious virus release in Fig 1A is striking (>500x at days 4 and 6?), however intracellular RNA differences are only observed at 6 days and is only 4-fold. While the later was still significant, is the results section down playing this too much by merely saying “both significantly lower”. Some perspective on the RT-PCR assay might help. What is the level of detection of this assay? This might say if the day 6 levels were the only ones quantifiable, and thus the lack of a difference prior to this point might not mean anything. Was a standard curve used, which would provide a more reliable means of absolute quantification?

A. This experiment was performed at a low moi infection (moi=0.01). That’s why HCV RNA levels remain low until day 4 post-infection. We have changed the y-axis to a logarithmic scale which illustrates better HCV RNA at the low level. The results showed that HCV RNA levels in every group were above the detection limit.

Q3. The blot In Fig 1D, actually all such blots involving detection of viral proteins, could use naïve cell controls.

A. Thanks very much for the suggestion. We performed a new experiment by adding a naïve cell control and presented this result in new Fig 1D of the revised manuscript.

Q4. When possible, the specific infectivity of virus should be described. This would be especially helpful to bring together the infectivity and RNA measurements described in Fig 1F and Suppl Fig S1C, respectively.

A. Thanks very much for the suggestion. We have calculated the specific infectivity and added the result in new Fig S1D. We also discussed the interpretation of this result in the revised manuscript (tracked version page 15 line 340 to page 16 line 347).

Q5. Quantifying viral proteins produced from subgenomic replicons by immunoblot is an acceptable means to compare RNA replication fitness, but it can be less sensitive to changes than other methods. RNA quantification would be better, but why not use replicons expressing luciferase genes, which are readily available?

A. We have shown the result of HCV subgenomic replicon RNA levels in glucose and galactose medium-cultured Huh7 cells (Fig S1B). This subgenomic replicon does not contain a luciferase gene.

Q6. This reviewer would prefer a slight variation to the cell-cell spread assay. Here, one could label the recipient cells with a fluorescent marker to be able to differentiate these from producer cells. This would allow one to differentiate between division of producer cells and actual cell-cell transmission.

A. This is a great question. To address this concern, we cultured the mCherry-expressing Huh7 cells (producer cells) mixed with naïve Huh7 cells (recipient cells). By seeding the same density of the cells (90% confluency) as we did in the HCV cell-to-cell transmission experiment, we found that the producer cells do not divide significantly during the 3-day incubation (see figure below). We have added the information of seeding confluency in the figure legend of the revised manuscript (tracked version page 35 line 866 to 867).

The cells were fixed after 3 days culture and stained with Hoechst (blue).

Red: mCherry-expressing Huh7 cells (producer cells)

Scale bar 100 μm

Q7. Just for clarification, what is the glucose/galactose composition of normal DMEM? Would modifying this ratio enhance the amount of virus researchers can generate in their HCV experiments?

A. The regular high-glucose DMEM culture medium contains 4.5 g/L (25 mM) glucose. There is no galactose in regular DMEM. We have not done systematic analysis by comparing different glc/gal ratio. However, we found that as long as excess amount of glucose is present in DMEM, HCV release will be no longer affected by supplementing galactose.
